# Unraveling the thread: understanding and addressing sequential failures in human-robot interaction

**DOI:** 10.3389/frobt.2024.1359782

**Published:** 2024-09-12

**Authors:** Lucien Tisserand, Brooke Stephenson, Heike Baldauf-Quilliatre, Mathieu Lefort, Frédéric Armetta

**Affiliations:** ^1^ Interactions, Corpus, Apprentissages, Représentations (ICAR) UMR5191, Centre National de la Recherche Scientifique, ENS de Lyon and Université Lyon 2, Labex ASLAN, Lyon, France; ^2^ University Lyon, Université Claude Bernard Lyon 1, CNRS, INSA Lyon, Laboratoire d’InfoRmatique en Image et Systèmes d’information (LIRIS) UMR5205, Villeurbanne, France

**Keywords:** human-robot interaction, in-the-wild, conversation analysis, sequentiality, grounding, contextualisation

## Abstract

Interaction is a dynamic process that evolves in real time. Participants interpret and orient themselves towards turns of speech based on expectations of relevance and social/conversational norms (that have been extensively studied in the field of Conversation analysis). A true challenge to Human Robot Interaction (HRI) is to develop a system capable of understanding and adapting to the changing context, where the meaning of a turn is construed based on the turns that have come before. In this work, we identify issues arising from the inadequate handling of the sequential flow within a corpus of in-the-wild HRIs in an open-world university library setting. The insights gained from this analysis can be used to guide the design of better systems capable of handling complex situations. We finish by surveying efforts to mitigate the identified problems from a natural language processing/machine dialogue management perspective.

## 1 Introduction

Until recently, robot systems have largely managed spoken interaction through controlled mechanisms such as finite state machines (e.g. Nakano and Komatani, 2020) and rule-based systems (e.g. Webb, 2000). These models, designed to carry out predefined tasks, can be quite rigid and commonly result in failures when confronted with the complexity of real-world situations. Unlike in controlled laboratory experiments, in open-world scenarios, it can be difficult to predict user behaviour. Users do not necessarily have a well defined representation of the robot’s purpose or the manner in which they should engage with it (e.g. Arend et al., 2017). And even when the purpose is understood, if humans “deviate” from the designed script/progression, for example by making two requests within a single turn or by returning to an earlier discussion thread, the state of conversation can become confused. New technologies, such as large language models (LLMs), may be able to offer increased flexibility in dealing with a larger context, a broader range of topics and varied formulations, however if their training data has only a limited amount of spontaneous spoken speech, they may struggle with the interactional elements of speech, such as turn-taking, which can differ significantly from written text or scripted media. Nor can LLMs alone incorporate instantaneous feedback (e.g., backchanneling) from the user to adapt their responses on the fly. Regardless of the underlying technology, it is important to understand the nature of speech and the underlying principles guiding real-life interaction in order to optimize system design to human behaviour. This motivates the current study which analyses HRI failures through the lens of conversation analysis (CA).

Previous studies based on CA have identified a major problem in the case of multi-party interaction where the meaning, timing and sequential positioning of turns addressed to the machine depend on the ongoing interaction between humans (Porcheron et al., 2017; Arend et al., 2017; Reeves and Porcheron, 2023; Gehle et al., 2015; Tisserand and Baldauf-Quilliatre, 2024). The issue is, these human-human interactions (from which the turns addressed to the robot depend) are complex, for this interactional complexity reflects the complexity of real-world social and cultural practices, roles, identities, etc. (Lund et al., 2022). Detailed studies on *temporally continuous* and *multimodal* interactions with machines also show that participants use specific practices when addressing a machine: these specific practices may index what they treat as an *adequate norm* for this purpose. For example, CA studies have identified the keyword formatting of turns (Pelikan and Broth, 2016; Avgustis et al., 2021) or vocal commands and unilateral departures (Licoppe and Rollet, 2020; Velkovska et al., 2020). In transposing turn-taking, similarities and differences have both been demonstrated (Pelikan and Broth, 2016; Majlesi et al., 2023). These results show that there is a need to explore in detail the practices transposed from human-human encounters and the new practices that emerge in a world where people are increasingly engaging with machines through speech and physical gestures in a sustained, temporally continuous manner.

Other CA-based studies have proposed models based on fine-grained analyses of human-human-interactions. For example, albeit not applied to *human-robot interactions*, Aoki et al. (2006) distinguished patterns in the conversational organization of schisming (i.e., the methodical accomplishment of splitting one conversation into sub-conversations (Egbert, 1997)), which could be detected to adapt the audio space in group meetings between humans. Multi-modal datasets of instructed laboratory interactions between humans (introducing themselves, playing a game, etc.) have been created with the hypothesis that they could be used for HRI (Tuyen et al., 2023). But only very few studies try to improve the design of a conversational agent/robot by drawing on the systematicity of the conversation analytic approach applied to the data (but see Lohse et al. (2009) for an example). Pitsch (2016) (involved in the previously cited collaboration) emphasizes the limits in *formalizing* interactions and thus, the need to carefully define what is worth being created in terms of *formal* objects that could be processed by the system. She concludes that the human capability to adapt to the machine can be leveraged by making transparent and explainable the robot’s actions.

This means that improving HRI requires finding 1) which human interactional norms are indexed (i.e. a reference made explicit *insitu*) and how it is adapted to the situation of using a robot, 2) which human norms are not made relevant, 3) which human norms are considered problematic by users during robot interactions, *e.g.,* because the robot is not a person, and 4) new norms relevant for interacting with a robot (that is, when people are interacting with a machine, especially in public, what are they expected to do with regards to the design of their verbal and multimodal actions. An empirical approach has the benefit of capturing displays of people making their conducts accountable (interpretable, justifiable, that can be attributed to their agency and intention) and with regards to norms of action such as the sequential norms of adjacency pairs that we present in Section 2.1. As we will show, this approach can evidence how acting according to a norm can be treated as a problem or treated as adequate, through the study of interactional phenomena like delaying, disalignment (Lee and Tanaka, 2016), repairing, accounts, etc.

In this paper, we try to respond to these challenge by investigating an in-the-wild scenario and analyzing what can be considered failures in the interaction with a social robot, based on the sequential organization evidenced by detailed analyses. In so doing, we uncover current limitations in the robot’s programming which should be considered in future designs. We focus principally on failures that are due to deficiencies in sequence organization. Indeed, one of the core findings of conversation analysis is the sequential organization of interaction: each action projects specific follow-up actions (indexing a normative *sequence of action*). The temporal unfolding is thus an important aspect when modeling interaction. We develop this dimension in the theoretical section (see Section 2.1). We show how some of the failures can be the outcome of humans transposing basic sequential organisation techniques that the program can not handle.

In the latter part of this paper, we turn our attention to possible technical solutions to the current shortcomings we observed. We also discuss the suitability of different dialogue management systems for the handling of the sequential flow in an open environment which includes non-elicited and non-guided interaction. Descriptive approaches to dialogue management have traditionally been used to handle focused service requests as they allow for precise programming and predefined responses tailored to specific tasks or inquiries. Generative AI approaches on the other hand can offer more flexibility for dealing with unexpected subjects, but their output is more difficult to control. The coupling of descriptive oriented approaches and generative AI remains a major challenge to be addressed in the coming years and is discussed in this article.

We first explain some of the theoretical concepts and methodological implications (Section 2) before presenting the analyzed data (Section 3). We then provide an analysis of some of the failures we identified (Section 4) and finally discuss which technical solutions could be applied (Section 5).

## 2 Theoretical underpinnings and methodological implications

In this section, we briefly introduce the sequential organization of conversations as evidenced by *Conversation Analysis* (*CA*) following a corpus-based approach (see Section 2.1). After that, we explain the methodological implications and the kind of knowledge we gain when applying *CA* to *HRI studies* that aim at improving human-robot interactions (see Section 2.2). Finally, we delineate what dimensions are involved in defining a failure with regards to the sequential organization of interactions, but also, by taking into account orientations taken by *HRI studies* (see Section 2.3).

### 2.1 The sequential organization of conversations: norms, context and HRI

In interaction, the context is seen by *Ethnomethodological Conversation Analysis* (hereafter *EMCA*) as a resource for interpretation by means of a mechanics of intention and expectancy (Levinson, 2006): prototypically, a question creates the expectancy for an answer. Through *sequence organization* (Schegloff, 2007; Kendrick et al., 2020) humans provide their interlocutor with the opportunity to show how they have interpreted a previous production (e.g., as a question, an offer, a response, an unexpected response). Thus, for a same turn produced by Pepper such as “How can I help you? Don’t hesitate to ask me what I can do”, participants can display that they interpret it as a directive (responding “oh okay then what can you do”) or as a proposal (responding with “thank you but you can’t help me”). These expectations of a next action are verifiable by the way others adapt to them (or not) in real time: the interaction is seen as a temporally continuous and incremental process and not a purely logical and serial one.

Although this sequential organization is operative at different levels of granularity and in different modalities, *adjacency pairs* account most strikingly for the accomplishment of such normative conducts and expectancy. The *sequence* of *adjacency pairs* is the relationship between two actions that are paired as types of action and accomplished in an orderly manner by (at least) two participants contiguously (Schegloff, 2007). A *First Pair Part* (e.g., a question) makes conditionally relevant a *Second Pair Part* (e.g., an answer), so that a response can be “officially absent” (Schegloff, 1968) in the continuous flow of interaction.

In the case for *human-robot interactions* and more generally interactions with *conversational user interfaces*, it has been evidenced that users transpose parts of the *adjacency pair* norms such as the type of next action made conditionally relevant (Pelikan and Broth, 2016; Reeves et al., 2018; Fischer et al., 2019; Licoppe and Rollet, 2020) and therefore conversation analysis could provide specifications for a system to handle such projections. This is why the present study focuses on *sequential failures* where a breach in conditional relevancy happens (see Section 4). In *HRI studies*, while sequencing between actions is very important for understanding the progression of dialogue, methods for modelling this phenomenon explicitly have only recently started to be investigated (Duran, 2023; Kunneman and Hindriks, 2022).

### 2.2 Methodological implications when applying conversation analysis to HRI

The *sequential* dimension of interaction is researched through the “*sequential*” *analysis* of transcribed data. This process can be summarized as addressing the question “why that now” (Schegloff and Sacks, 1973) each time an accountable action is produced by an interactant. This question is posed at each stage of the interaction (turn-by-turn, second by second). Paying attention to the details of the temporal unfolding of turns that are exchanged between two participants allows the researcher to evidence their relationship with norms of action that are indexed and can be distinguished (Muhle, 2024). When analyzing *human-robot interactions* with this theoretical and methodological framework, some insights can thus be provided so that the designer and the software engineer can decide on alternative choices that have an impact on the robot’s output, with the aim of aligning to the users’ orientation towards the norm they make noticeable (Pelikan et al., 2024).

For example, below is the detailed transcription of several users’ response to the robot’s greeting and/or offer extracted from the corpus used in the present study:

220324_48------------Case 1

01 robot:  hello (.) je peux t’aider/

eng     hello (.) can I help you/

02         (0.8)

03 human:  ah oui

oh yes

220309_84------------Case 2

01 robot:  coucou\ (.) je peux t’aider/

eng     hi         can I help you

02         (0.5)

03 human: euh: oui/

eng    uh: yes/

220929_17A-----------Case 3

01 robot:  hello (.) moi c’est pepper (.) je peux t’aider/

eng     hi  (.) my name is pepper (.) can I help you

02         (0.9)

03 human: euh: oui/

eng    uh: yes/

220324_84------------Case 4

01 robot:  salut (.) je peux t’aider/

eng     hi (.) can I help you/

02         (1.1)

03 human1: oui:

eng     yes:

04         (0.2)

05 human2: tu veux lui demander quoi/

eng     what do you wanna ask/

06 human1: ché pas

eng     dunno

220926_28------------Case 5

01 robot:  hello (.) moi c’est pepper (.) je peux t’aider/

eng     hi  (.) my name is pepper (.) can I help you

02         (0.3)

03 human: euh: oui je: je voudrais: j’ai besoin de ton aide

eng    uh: yes I: I wou:ld   I need    your help

04 human: comment (0.6) peux-tu m’aider/

eng    how  (0.6) can you help me/

220324_48------------Case 6

01 robot:  je peux te donner des infos ou t’orienter\

eng       I can give you informations or orient you

02         (1.7)

03 human: ben::j::‘ veux::: (1.1) j‘ vais prendre un café s’il vous plaît

eng    well:: I:: will::: (1.1) I’ll have a coffee please

While such cases can be seen as a collection of acceptances (Cases 1–5) and an isolated case of request (Case 6), they can also be seen as a collection of the normative adjacency pair [offer → acceptance | reject | request] being indexed as problematic and not fully applicable, thanks to the detailed account of events that occurred. Indeed, the gaps (Cases 1–4 and 6), the hesitation markers (Cases 2–3 and 5–6) and the voice lenghtenings (Cases 2–6) show that the humans do not treat the robot’s offer as preferably projecting first and foremost an acceptance or a direct request (Pomerantz and Heritage, 2012; Schegloff, 2007). More drastically, in Case 1, the *change-of-state token* (Heritage, 1984) even displays that having to participate in such a sequence is surprising. Finally, other accounts also inform us about the users’ relationship to the offer sequence (Schegloff, 2002; Heritage and Watson, 1979). The account asked by human2 to human1, who accepted the offer (Lines 5-6 of Case 4), but also, the return question that initiates the repair of the meaning of a previous acceptance (Line 4 of Case 5) show that this action–the acceptance of the initial generic offer–is not treated with the same social implications as the actual actions accomplished through the offer sequence as a resource (Kendrick and Drew, 2014a). If we build a collection of cases that all include one of these details (especially the resources that delay the acceptance), then such a collection can quickly grow as such patterns are common and not surprising in this situation where average users do not know the purpose of the robot.

When these results are oriented towards HRI, drawing attention to the fact that these generic initial offers appear as inappropriate can then lead the designer to change the scenario, for example, by completely removing this robot action at this moment, and instead, proposing an alternative (i.e. the scenario does not require that the user actually needs something), if one wants to align to the norms that the users orient to or not. Or, having shown that phenomena such as voice lengthening, delayed answers or hesitation markers regularly happen in a localized context (this generic initial offer can be accounted as inappropriate by the humans), one can also decide to build a system that can specifically handle such localized phenomena (see Section 5.2) for these can be responsible for sequential failures (as shown in Section 4.3). Furthermore, the resources identified can be treated as cues, systematically annotated, and queried in the corpus in order to identify other contexts of inappropriate expectancy. What matters is to make an explicit and justified decision with regards to such results, as we explain in Section 4.

Between a human and a robot in a public space, the presence of other humans (hypothetical or verified) raises, for the users, the practical issue of producing conducts that are also adequate from the point of view of other humans. Thus, in such situations, specific practices for exchanging turns with this machine are made relevant, monitored and aligned on as a norm oriented to by the users. *HRI studies* are mainly based on experimentation in laboratory settings. This type of setting biases the orientation towards norms (Pitsch, 2016). When users aim to complete a specified script or activity within experimental boundaries, they also align with the experimenter’s expectations. This means they might persist through the robot’s failures, rather than solely responding to the interaction’s emergent goals with the robot. We therefore claim that research based on real-world situations is crucial to improve the design of user interfaces based on conversational dynamics and social robots tailored to it. Naturally occurring interactions or in-the-wild experiments (where users can act–or not–with the agent the way they want) are necessary to understand what is treated by humans as failures with regards to norms in the interaction that can only be relevant in such a context.

With regard to the relationship to human-human interactional norms when interacting with a Pepper robot in the public space, it has been shown elsewhere that users account for a deviation when transposing other social norms of interaction. For example (Licoppe and Rollet, 2020, p.172) evidenced that when the robot responds appropriately and is successful in achieving basic sequences of action, the fact that this performance can be assessed as surprising or pleasant by the humans not only deviates from a human-human normal and subliminal expectancy (Enfield and Sidnell, 2021), but it also indexes the fact that the robot is first and foremost treated as not being a competent interactant. When it is the case for the humans to respond appropriately, it has been shown that they also account for a deviation by the means of systematically treating as humorous the fact that they have to treat the offer as implying *preference organization* (i.e., acceptance are more straightforwardly produced than rejection that are more elaborated), when producing the *offer reject* (Tisserand and Baldauf-Quilliatre, 2024).

### 2.3 The collaborative definition of a failure

Previous epistemological reflections on research designs bridging *EM(CA)* and *system design* (Button, 1990; Button and Sharrock, 1995; Dourish and Button, 1998; Dourish, 2006; Pitsch, 2016; Pelikan et al., 2024) have emphasized the need to make explicit the rationalization used when CA results are applied to system design (e.g., in the form of *design implications* or so-called *cues*) and for what situated practical purposes. When addressing such potential collaborations, useful observations have been drawn with regards to what it really means to attempt to model human interaction by transforming into rules the norms identified by CA. Firstly, a rule-based system cannot be compared with human interaction (Button, 1990; Button and Sharrock, 1995), nor can a statistics-based speech system (Schegloff, 1996, p.22). Indeed, a human may expect from a machine a set of appropriate answers, that is, answers that can only comply with a particular expectancy. From another human, one may always treat an answer as *accountable*, that is, the fact that the respondent had a reason to do so in this way (Enfield and Sidnell, 2021). Secondly, when “indexical expressions” (indexicality is the essential property of human accountable conducts whose meaning can always be negotiated through the mechanics explained in Section 2.1) are transformed into “ideal expressions” (with a self-contained definite meaning/purpose), they participate in a structure missing this accountability (Garfinkel and Sacks, 1970, p.339). As these observations were oriented towards goals, in the present study, we draw on the paradigms they propose in order to redefine our goal, which is the enhancement of “facilitating” human-robot interaction rather than “reproducing” naturally-occurring conversation.

Thus the enhancement is explicitly measured through variables that do not account for the machine’s conversational competence in a natural sense. In our case, *cues* are identified in the same vein as “ideal expressions” that can “enhance” the human-robot interaction from an *HRI* perspective. Our rationale for envisioning such enhancement is the fact that the specific practices identified by CA can be seen as the users’ orientation towards a rule-based system (the timing in turn-taking management, keyword formatting, expecting that the agent has only one possible answer, etc.), and this approach is in line with *HRI studies* which demonstrated that the users’ acceptance of a robot may not be based on human resemblance (Ghosh and Ghosh, 2021; Nazir et al., 2023).

Drawing on these previous work, in this paper, a “failure” is seen as the intersection of four dimensions that are made explicit:

1. What the humans and the robot do with regards to interactional norms: whether they transpose, problematize, or orient towards a machine adequacy, as explained in Section 2.1,
2. If and how the participants treat what happened in the first dimension as a failure or a problem for the ongoing interaction,
3. What the system did,
4. The fact that methods exist in order to handle the issues identified in (1) and (3), that is, the failure is “computable” (or it can be handled by design practices).

A sequential failure occurs in the first dimension when conditional relevancy is breached between the human and the robot and when the sequential organization of conversation is involved in such a failure. Conditional relevancy is breached when:

1. The robot produces an unexpected type of response or no response at all when it should
2. The robot takes the turn when it should not or
3. The user does not understand that it was his/her turn and that an action was expected.

Note that the absence of a response followed by a self-repair from the robot (such as “sorry I did not understand”) is not a sequential failure as it accounts for the missing response and potentially initiates a repair from the human. Here, the role of Conversation Analysis is to provide the analysis of the first and the second dimensions, thereby highlighting what type of norm-related problem exist. Thus it may justify the importance of success of an *HRI* approach that solved a particular problem that can be handled. However, the humans may treat merely as a failure (second dimension) what *HRI* can identify as a problem that can be resolved.

Furthermore, the second dimension might help categorizing the events as explicitly not a failure at all by analyzing the users’ orientation towards purposely putting the robot into a failure situation, which is a pervasive situation that we might term (albeit ironically) a “successful failure” from the user perspective. This perspective can be useful, outside the laboratory, when such a failure can not be handled by state of the art *HRI* and *NLP* solutions. We present these four dimensions to emphasize the fact that choices are made according to the perspective (user or designer) adopted (Pelikan et al., 2024).

## 3 The data

We decided to investigate a service-encounter setting since it provides a rather simple and strongly normative, asymmetrical sequence organization (asymmetry of roles, turn allocation, needs, lexical choice, etc., see Drew, 1992; Heritage, 1998), where we can expect humans to draw on everyday sequential mechanics of conditional relevance (e.g. requests/offers and its acceptance/refusal, see Drew and Couper-Kuhlen, 2014). But also, it is a plausible purpose for a commercial robot like Pepper, and this attention paid to the authenticity of commercial robots used in public places explains why its initial programming is based on its built-in functionalities only. The situation has been designed so as to reproduce the typical way an institution (in this case, a university library) showcases a robot (it should serve a purpose, the services that it provides must be doable by a human, it must not disturb the environment). This setting allowed us to test if users spontaneously treat the robot’s turns (verbal turns as well as bodily orientations) as actions demonstrating participation in a regular desk service encounter that they have to adapt to the robot, without further guidance, contrary to settings in a public space where robots greet the users and propose an activity with a limited set of answers (e.g. Ben-Youssef et al., 2017; Gehle et al., 2015). In the present study, users were passersby, users of the library, that had not been recruited beforehand.

We chose to use a descriptive model for this study to cover the scope of possible domain-specific information and actions requested by the users. The program (QiSDK^1^) recognizes keywords that trigger a state machine to select a state specified on the diagram of transition states (this is performed through a matching function). This design choice is subject to the inherent limitations in descriptive approaches, but allows us to keep the application under control and prevent problems coming from more black box approaches (e.g., AI generative models). While this model may not represent the current state of the art, it is sufficient to produce analyzable data and to later extend the model, bearing in mind that each of the two approaches (descriptive-based versus generative-based) has its own specific drawbacks (discussed in Section 5.1). Moreover, the failures we will discuss go beyond the limits of the descriptive model used, as it is the human recurrent practices that we focus on.

**FIGURE 1:**
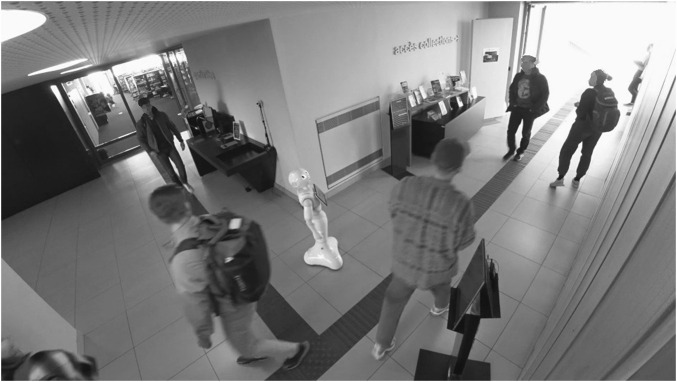
An image from one of the large angle cameras, demonstrating the placement of Pepper in the library entrance where people are passing by. The reception desk is 3 m away on the left, outside of the frame.

The robot was placed in the vicinity of the reception desk, at the entrance of the university library (Figure 1). As the robot was not programmed to move (only to rotate), it was easier to define a recording area. Two large angle cameras were placed in order to capture the whole scene and especially to understand how users approached the robot before the opening of the interaction since it might be important for its unfolding. We also recorded the audio and video streams from Pepper’s tablet.

With regards to personal data protection, posters were placed near the various entrances of the library. After each interaction, a team member obtained signed consent, otherwise the data was deleted. Eleven recording sessions took place, leading to approximately 9 h of human-robot interactions (N = 730). A subset of them, those where consent was specifically given for online sharing for research purpose, will be made available in the future.

The data have been transcribed, time-aligned and annotated with regards to adjacency pair norms and repair practices (Tisserand et al., 2023) in ELAN^2^ according to ICOR^3^ conventions. The analysis allows us to identify the following (typical) failures that we describe in the subsequent section.

## 4 Sequential analysis of failures

Our analysis here highlights four typical situations which are difficult to address for conversational agents:

1. When a human’s embodied turn refers to a pervasive social practice (Section 4.1),
2. When the human takes back the sequence initiative (Section 4.2),
3. When the construction of a turn is not straightforward (Section 4.3),
4. When the user orients towards multiple actions at the same time (Section 4.4).

For each type, we will first present transcripts coming from the corpus. We will then explain what are the sequential moves that humans orient to in order to conduct their interaction with the robot (first dimension) and how these led to the sequential failure. We point out what caused the failure in the current dialogue system and identify the types of cues that could be used in order to prevent such failures. Modelling techniques that can be used to improve the handling of these cases are then reviewed and discussed. These approaches seek to introduce greater flexibility into dialogue systems that need to be able to handle turns in interaction with turn-taking cues, multiple actions/intents and parallel threads. Thus, the conversation analytic part is completed in Section 5 by dialogue modelling proposals from the state of the art to address the drawbacks.

### 4.1 Multimodality and sequentiality with reference to ordinary activities

Here, the failure is the fact that the greeting turn produced by Pepper orients towards the recognition of “opening an interaction” while the greeting accomplished by the human indexes another type of “greetings alone” activity. Thus, at the end of Line 3, Pepper is accountable for making a response conditionally relevant (responding to its offer) while the human prevented this possibility in the first place:

220929_10

((human is passing by))

01>human:   salut Pepper (0.2) bonne journée/

hello Pepper (0.2) have a nice day/

02>         (0.8) ((human is leaving))

03 robot:   hello (0.2) moi c’est Pepper (0.2) j‘ peux t’aider/

hello (0.2) my name is Pepper (0.2) can I help you/

Here, the robot (Line 3) produces a greeting turn orienting towards the continuation of the interaction (greeting + self-presentation + offer) instead of a “greetings alone” interaction. Activity-wise, the apparent opposition (“hello” vs. “goodbye”) (Line 1) is actually the accomplishment of an ordinary practice among humans. In settings such as workplaces, where people routinely see each other for the first time of the day, members perform “greetings only” interactions without stopping to walk (Tunser, 2014, pp. 121–126). This is what the human is trying to perform with Pepper in the example above. Categorization-wise, he treats the robot as being part of his routine at the university library, which is also indexed by the address term “Pepper” packed with the greeting.^4^

With regard to the *FSM* programming, the failure is that it recognized the “hello” (“salut”) greeting keyword only. However, as shown in Section 5.3, multiple actions in turns are recurrent, this specific case of “greetings alone” is pervasive when a robot is placed in a high-traffic public space (see additional cases). Typically, this kind of failure cannot be reproduced in a laboratory setting. It is not a failure from the point of view of the user who continues on his way^5^, however, it is a failure in this setting as the robot has been activated and is waiting for a next turn: thus problems may arise with other passersby in the immediate future. Ideally, the robot would have produced a reciprocal terminal (e.g. aligning on the same words “have a nice day” Schegloff, 2007, pp.195–207) or greeting exchange. Still adequately, the robot could also not respond at all, as the user, who is leaving, looses the opportunity to treat the absence of response as a breach in conditional relevancy.

As a workaround to the unavailable meaning of this turn as participating in a social practice (distinguishing two activities), cues are made available by the human so that these can be used by a machine in order to overcome such sequential failure:

• The action-types [greetings + terminal exchanges] in the same turn (that can be handled by multi-threaded approaches, see Section 5.4);
• The humans produces this turn while continuing their walk (that can be handled by “non-verbal” cues approach/exit, see Section 5.5).

The first case that we presented emphasized the “greeting alone” activity by the two-action formatting of the human’s turn and the robot’s response. The more common cases depend on the visual cue: humans stay oriented towards continuing their walk through a torque of their body (Schegloff, 1998), that is, the lower part of the body is not oriented towards the robot while the upper part is. Below are variant cases of humans continuing their walk while producing a greeting, which are prone to the failure identified and can be processed through visual cues:

220318_16----------------------

((two humans are walking across the corridor))

01 human1: (inaud.) ((slows down in front of Pepper and waves))

02 robot : hello\

((humans laugh and leave, Pepper waits for an answer))

220324_15----------------------

((four humans are walking across the corridor))

01 human1: wesh ((waving))

((humans continue their walk))

220325_63----------------------

((two humans are walking towards exit))

01 human2: ((approach PEP))

02 human2: sele:m xx ((waving and continuing his walk))

03       (5.7) ((human2 stops and torques towards robot))

04 human1: wesh negro/ ((waving))

05          (1.6) ((human leaves and laughs))

06 robot:  salut (.) je peux t’aider/

eng     hello (.) can I help you/

((humans continue their walk, Pepper waits for an answer))

220321_21----------------------

((two humans are walking across the corridor))

01 human1:  bonjou:r ((towards Pepper and continuing her walk))

eng       hello:

02          (0.7)

02 robot:   oui/

eng      yes

((humans continue their walk, Pepper wait for an answer))

220328_06A---------------------

((human is walking towards exit))

01 human: (2.2) ((slowly stops her walk and stayed torqued towards Pepper))

02        (1.0)

03 human: (1.6) ((one step forward while staying torqued towards Pepper))

04        (1.5)

05 robot:  salut (.) je peux t’aider/

eng     hello (.) can I help you/

06 human: ((laugh and leave))

220329_48----------------------

((three humans are walking across the corridor))

01 human1: bonjou:r ((towards Pepper))

eng     hello:

02         (0.6) ((Pepper raises head while humans continue their walk))

03 human2: bonjou:r ((towards Pepper))

eng     hello:

04         (1.4) ((humans continue their walk))

05 human2: c’est marrant/

eng     that’s funny/

((humans continue their walk))

220329_80----------------------

((four humans are walking across the corridor))

01 humans: ((talking together))

02 human1: (1.4) ((slows down and wave at Pepper))

((humans continue their walk))

220331_03----------------------

((two humans are walking across the corridor))

01 human1: ah: (0.4) mec

eng     oh:       boy

02 human2: bonjour ((towards Pepper))

eng     hello

03 human1: bonjour ((towards Pepper))

eng     hello

((humans continue their walk))

220331_32----------------------

((four humans are walking across the corridor))

01 human1: bonjou:r

eng     hello:

02 human2: ((laughter))

((humans continue their walk))

220926_38B---------------------

((two humans are walking across the corridor))

01 human1: wesh mon (gros) (0.3) bien ou quoi/

eng     hey my boy      doing good or what

02         (0.6) ((humans continue their walk))

03 robot:  tu cherches quelque chose/

eng     you looking for something

04 human2: ((laughter))

05 human1: non (.) j‘ te dis bonjour juste ((torqued towards Pepper))

eng     no I’m just saying hello

06     (1.9) ((humans continue their walk))

07 human1: i‘ m’a pas répondu pepper\

eng     it didn’t answer pepper

220929_12---------------------

((human is walking across the corridor))

01 human1: hi: ((towards Pepper and continuing his walk))

02       (0.8) ((human continue his walk))

02 robot : salut (.) moi c’est pepper (.) j‘ peux t’aider/

eng      hi my name is pepper can I help you

((human continue his walk))

### 4.2 A response coupled with an initiation: When the user takes back the initiative

Another typical case that leads to failures when the system allows only one decision (one change of state) is the case where the user responds to the current state expectation as a second pair part of a sequence of actions and then, in the same turn, the user initiates a new sequence. Inevitably, if the robot succeeds in parsing the second pair part placed in first position and the next state is initiated with a turn, this will lead to an overlap. This is caused by a next action that does not take into account the full turn produced by the human, such as in the two cases below:

220317_05

01 robot: je peux te donner des directions ou des informations sur la bibliothéque\

eng    I can give you directions or information about the library\

02 robot: (.) ça t’intéresse/

eng    (.) are you interested/

03>human: oui/ (.) je veux un [café]

eng    yes/ (.) I’d like a [coffee

04 robot:        [okay\ comment est-ce que je peux t’aider/

eng          okay\ how can I help you/

05 human: où sont les cafés/

eng    where is the coffee/

220317_29

01 human: à quoi tu sers/

eng    what are you for/

02    (0.8)

03 robot: je peux te donner des directions ou des informations sur la bibliothéque\

eng    I can give you directions or information about the library\

04 robot: (.) ça t’intéresse/

eng    (.) are you interested/

(0.3)

05>human: oui (0.5) [où sont les toilettes/

eng    yes (0.5) [where is the bathroom/

06 robot:     [okay\ (.) comment est-ce que je peux t’aider/

eng      [okay\ (.) how can I help you/

07         (0.5)

08 human: où est la machine à café/

eng    where is the coffee machine/

In these two cases, the program processed the human input “yes” and provides a response to this case of offer acceptance alone, instead of treating the succession of the two actions [acceptance + request]. In a service encounter scenario, the response to the initial offer “can I help you” is prone to such situations as the initial, generic offer is projecting the immediate formulation of a request (Kendrick and Drew, 2014a) in such an institutional setting (Lindström, 2005, p.213). This offer can also be verbally accepted as the first part of the turn and hereafter, in the second part of the turn, the expected request is formulated.

With regard to the goal-oriented task, the difficulty here is the fact that both cases (acceptance alone, or acceptance + follow-up action) must be discriminated between at this place. In 190 interactions that have been systematically annotated with regards to adjacency pairs, 81 acceptances have been identified, of which 54% (N = 44) appear in a turn with a follow-up action, while 46% (N = 37) are produced alone. Among the turns produced with a follow-up action by the human, 17 are produced with no pause or a micro-pause (12 questions/requests, 2 directives, 1 account, 1 self-identification), and 27 are produced with a pause of average 0.7 s (19 questions/requests, 6 directives, 2 accounts, 1 howareyou-sequence).

As already pointed out by (Skantze, 2021, p.13), a silence *ad hoc* rule would not be a solution. Here we provide additional reasons for this: as we can see in Line 5 of excerpt 220317_29, a silence threshold would need to be longer than (0.5) seconds, which is a long time from a conversational norm standpoint. Especially, when a human responds with “yes”, the turn produced by Pepper can therefore be retrospectively treated as a (pre-) proposal from the robot, the “yes” being a *go-ahead* addressed to the robot, expecting an immediate new turn from it (a proposal).

Here, no specific verbal cue can be detected to prevent this failure. As such, with respect to norms of adjacency pairs, it is important to identify such situations. However, this overlap-proffering situation shows how important it is to have a system that is able to process overlap and/or a system that can continue to listen to possibly future talk, which Pepper’s built-in software does not (also evidenced by Pelikan and Broth, 2016). Overlap in conversation can be treated ordinarily (Schegloff, 2002; Jefferson, 2004). In HRI they can be managed casually, but they can also be addressed more critically when they result of breaches in normative expectation (Majlesi et al., 2023). Here, from the point of view of the humans, a conventional repair practice tailored for HRI (like word selection, see Stommel et al., 2022) is produced in order to manage the failure.

### 4.3 Incomplete turns with turn-holding device and repairs

The cases below are more commonly studied than those in the previous sections (e.g. Skantze, 2021; Baumann et al., 2017). These are turns produced by humans with turn-holding devices (Line 6 in the first excerpt and Line 4 in the second excerpt below). The observable sequential failure is the overlap between the human and the robot’s turn: that is, the robot should not respond when it does (moreover the robot’s turn is inappropriate). This failure stems from sequential organization as in each case, the human displayed that the turn was taken and that the action would be completed. They thereby provided the interactional work of displaying their alignment on conditional relevancy and the robot did not take this into account.

220324_79

01 human1: [oula (.) i‘ m’a vu

eng     [wow (.) it saw me

02 robot:  [ouais\ (0.2) je peux t’aider/

eng     [yeah\ (0.2) can I help you/

03         (0.3)

04 human2: ((laugh)) (0.8) ((laugh))

05         (0.4)

06 human1: euh::: (0.8) c’est où/ la:::    (0.8)

eng     hu:::m (0.8) where is/ the::: (0.8)

07 robot:  je peux t’orienter vers différents [endroits de la bibliothéque\

eng     I can orient you towards different [places in the library\

08 human1:               [((laugh))

220928_56

01 robot:  tu cherches quelque chose/

eng     are you looking for something/

02         (0.2)

03 human1: oui

eng     yes

04 human1: je cherche les euh: (0.3) le rayon [informatique

eng     I’m looking for uh: (0.3) the section [informatics

05 robot:                [tu as dit que tu voulais aller où/

eng                 [where did you say you wanted to go/

06         (0.5)

07 human1: le rayon ↑informatique

eng     the section ↑informatics

From the point of view of the state machine, each time, what happened is the program found a match for the repair list of words: it recognized “where is” (Line 6 in first excerpt) and “I’m looking” (Line 4 in second excerpt).^6^ However, the problem can be generalized as having the robot processing as complete what is, in fact, an incomplete turn designed as such. What the human is doing here is actually following the norms of conditional relevancy given the *First Pair Part* produced by the robot: he acknowledges the fact that a request is expected (with the “yes” Line 3 in excerpt 220928_56), and that it’s his turn (that is taken with the turn-initial hu:m in excerpt *220,324_79*.^7^

Besides the user’s display of alignment, turn-holding devices are also used within-turn: these are the filler “hu:m” Line 4 in excerpt 220928_56 and the voice lengthening on the determiner “the” Line 6 in excerpt 220324_79. The fact that the robot does not align on the use of such turn-holding devices is the source of the failure, as the user claims the right to use these. Furthermore, within-turn silent pauses are made relevant by the use of such devices. Ideally, if the robot was more responsive and could handle overlap, a more subtle repair initiation (such as “huh?”) could be used in place, anticipating the possibility of an overlap. As said in Section 4.2, overlap among humans is not a phenomenon to strictly avoid but to manage in real time, and so is the negotiation of turn-ending (Schegloff, 2002). Therefore, such online small feedback from the robot may be interpreted by the users as relevant in both cases (complete turn or incomplete turn). Online non-verbal feedback has been tested successfully in *HRI* in order to help generating the expected input (Pitsch et al., 2013).

The situation in which a robot is placed in a public place where users do not know its purpose and its functioning make these turn-holding devices ubiquitous. The cues made available by the human in order to overcome such sequential failure are the following:

• The early display of alignment by the user: the (pre-) beginning of the turn shows that the user will provide the type-conforming answer;
• The recognition of turn-holding devices that are used;
• Syntactic completion.

However, syntactic completion would not be a useful cue in the case below. The human, this time, is re-enacting its turn as an overlap repair:

220928_19

01 human1: bonjour peppe:r

hello peppe:r

02         (1.9)

((laughter))

((human1 leans towards pepper))

03 human1: [où

[where

04 robot:  [tu cherches quelque chose/

[are you looking for something/

05         (0.9)

06 human1  [euh: ]

[uh: ]

07 human2: [l- l’ét]age (0.4) l’étageavec le s[port/

[the flo]or (0.4) the floorwith s[port/

08 robot: [pour te rendre aux étagessupérieurs

[in order to access upstairs

((robot continues))

Here, the syntactic completion criterion is validated with “l’étage” (which can be translated as either “the floor” or “upstairs”). The state machine can provide an answer to the question “how to get upstairs”, and that is the response that is triggered here. But the program should also be designed to provide the floor number associated with subject-based sections (such as the floor number where the sports books are located). It is this second type of request that the human asks the robot (Line 7). Here, the robot failed in that it processed the chunk of audio delimited by the 0.4 silence (Line 7) and classified it (as a “how-to-get-upstairs” question) while the turn was actually continuing (as a “subject X floor” question).

Here, what leads to the formatting of the request on Line 7 is that two humans are competing for the floor in order to produce the request. After the robot’s offer (Line 4), human1 claims the right for the turn with the “uh” (Line 6) as he leans towards Pepper (from Line 2) and already tried to formulate a request (Line 3) after the greeting (Line 1). Human2 also claims the right for the turn in overlap (Line 7). The silence of 0.4 s in the middle of his turn is the repair of his overlap with human1. Thus, such silences can be classified as an overlap repair if the information that “two humans with different voices are talking at the same time” is detectable.

Therefore, the cues made available by the human in order to overcome this type of sequential failure are the following:

• The initial (multimodal) display of alignment by the users (as above);
• The recognition of overlap (among humans) as the context for the turn processing (the function of the silence).

### 4.4 Two possible next actions

In this last case, the sequential failure is very simple to identify: the robot did not provide an answer (Line 7) to what might appear to be a simple move by the speaker (an offer acceptance). The robot was programmed to recognize the “yes” word in this state, however this word (Line 6) is part of a multi-unit turn (not separated by gaps) that plays a role in the sequential organization of such an opening. This raises a practical problem with regard to sequentiality: once identified, the initiating actions cannot be treated as a batch process of queued tasks. In 190 interactions that have been systematically annotated with regards to adjacency pairs, 163 pair parts (out of 1,417) are produced while there already is another expectancy for sequence completion. This is the case in the transcript below.

220321_06

01 robot:  coucou\ (.) je peux t’aider/

eng     hi\ (.) can I help you/

02         (0.3)

03 human2: .tsk .h coucou\

eng     .tsk .h hi\

04 human2: [((laugh))]

05 human1: [((laugh))]

06 human2: tu vas bien/ oui tu peuxm’aider:\

eng     how are you/ yes you can helpme\

07        (1.5)

08 human2: si tu m‘ réponds pas

eng     if you don’t answer me

09      (2.0)

10 robot:  comment est-ce que je peux t’aider/

eng     how can I help you/

In Line 1, Pepper produces two *First Pair Parts* in the same turn: a greeting (“hi”) and a generic offer (“can I help you?“). This packing of two First Pair Parts is relevant and common in desk service encounter openings. These actions project two conditional relevancies: a return greeting, but also an offer acceptance OR rejection, OR some request/question (Kendrick and Drew, 2014a, p.101). In Line 3, the human produces the projected second greeting. That is, at the end of the turn Line 3, there is only one projected action (a response to the offer) that is still relevant. After the laughter (Lines 4–5), the human produces (Line 6) a non-projected new action, a *First Pair Part* in the first segment of her turn (“how are you?“) which creates a new projection (a reciprocal response). Within the same turn, the projected offer acceptance is finally produced (“yes you can help me”). At this point (end of turn Line 6), the Second Pair Part made relevant by the offer is accomplished, but relevantly for a service encounter, another sequence is now projected either from the robot initiation (a proposal or second offer), or the human may now produce a request.

This means, that in Line 7 during the silence, two courses of action are again ongoing, with two types of projections on the next turns. On the one hand the “how are you” in l.6 projects a second pair part that is a reciprocal *howareyou* (Schegloff, 2007, pp.195–202). On the other hand, the overall structural organization makes relevant a second offer/proposal from the robot or a request from the human. The return “how are you” is more relevant than the second offer as its relevancy is settled by a first pair part (Stivers and Robinson, 2006).

The utterance on Line 8 designates the preceding silence as a failure. Thus Pepper has the right to treat it as the initiation of a repair sequence (in other words, a correction of the previous turn/action), which again makes conditionally relevant a repair completion. Also, at this point in the unfolding of the interaction, ostensibly the human has withdrawn the possibility to produce the request herself (as hypothetically expected from service encounter norms), as the turn-allocation to Pepper is reinforced. On Line 9, another long silence shows that the human maintains the possibility for Pepper to produce any next action that could align with the possibilities that we pointed to. On Line 10, finally, Pepper produces the possible (among other actions) second offer, designed as a question that more clearly pursues the production of a request from the human. At this point, the repair is completed.

As we can see, the sequential organisation of interaction can imply different possible next actions. The embodiment of such actions in time has a great impact on the interaction. Here, what can be leveraged from sequential organization in order to process such talk-in-interaction is the fact that:

• There are two actions in the same turn (without even a small pause);
• A “how are you” is relevant after the greeting exchange;
• The fact that possible next actions, once identified, can be ordered: the expectancy is not the same turn-to-turn, sequence-to-sequence, with regards to overall structural organization.

The fact that the interaction order is operating at multiple levels of granularity is also the source of error in the following excerpt:

220317_24

01 human: where can I find books about math?

02 robot: ((provides information as aresponse to the user’s question))

03 robot: is that clear?

04 human: yes thanks

05 robot: okay, I will repeat ((pepperrepeats turn L.2))

Here, the robot recognized “no thanks” (l.4)^8^: it thus repeats the answer to the user’s question (l.5). Differentiating “no thanks” vs. “yes thanks” is difficult for an ASR in a noisy environment. But these two possibilities are not equal with regards to what it accomplishes in the interaction. The “no thanks” accomplishes one action (a rejection registered as such). Here again, what is relevant is the ordering of possibilities: the “thanks” retrospectively indexes and expands the previous question/answer sequence (ll.1–2). The human first answered “yes” to the confirmation request (l.3) but also thanks the robot for its service (provided l.2) at the first possible slot.

## 5 Towards natural human-robot interaction

To overcome (some of) the limitations observed in the previous sections (such as speaking out of turn (Section 4.3) and the shallow/insufficient treatment of both two-action turns (Sections 4.1 and 4.2) and multiple active adjacency pairs (Section 4.4)), we require a model that can 1) make more sophisticated use of turn-taking and turn-holding cues, 2) identify more than one action within a turn, 3) learn to handle multiple threads in the interaction and 4) incorporate multi-modal features into its context understanding. In this section, we will delve into each of these requirements and review strategies that have been proposed to address these complexities as best as possible.

The limitations so described and the way to address them is very much affected by the ability of a dialogue manager to capture the flow and meaning of conversations. Indeed, if a model can comprehend these factors, it can easily 1) infer the contextual end of turns, 2) & 3) identify actions and threads that are not associated in a random way but aligned with the meaning of the conversation.

In dialogue systems, the current state of the discourse can be modelled using rule-based, frame-based or end-to-end systems. Rule-based and frame-based approaches are more directed toward the expertise of the human designer, while end-to-end systems are based on statistics extracted from a large corpus of texts and can be refined or oriented thanks to a pre-prompting strategy in order to fit the application needs. In this section, we first introduce and discuss these two popular families of methods to catch the contextual meaning and discuss inherent drawbacks. We then cover the approaches from the literature that can mitigate the limits described to a certain extend. Many of the approaches are hybrid and borrow both from hand-made settings and automatic learning.

There are of course other limitations that prevent smooth interaction between Pepper and the users, for example computational latency and the noisy environment which degrades automatic speech recognition. In this work, however, we choose to focus on the elements pertaining to turn-taking and conditional relevancy.

### 5.1 Contextualisation as a support for human-robot interaction

#### 5.1.1 Handcrafted approaches

As a task-oriented conversational agent, the robot is set up to provide answers for each targeted question. One way to address functionality is rooted in reproducing what is expected to be seen, to mimic what a human would do. The general principle being that the model contains expert knowledge which guides the behaviour of the system. This can be done thanks to a set of human-made rules designed to make decisions and provide solutions for specific problems. It can also include other specific supports, such as graphs, to map interactions.

This family of approaches can offer numerous benefits, such as intelligibility and easy updating. They nevertheless suffer from drawbacks including their inability to handle unexpected situations that have not been modelled. It is very difficult to model all the sequences that can occur by hand, especially when it involves semantics and conversations. In our experiments, interactions can easily exceed the scope the system is prepared for. Many of the approaches covered below follow the same logic, in particular those used in multi-thread modelling (Rosé et al., 1995; Lemon et al., 2002; Klüwer, 2015; Maraev et al., 2020). Finite-state machines (FSM), like what is used by Pepper, belong to this category.

Complications ensue in FSMs because the interaction does not always adhere to a strict set of succinct linear moves. We see this when the user performs two actions within the same turn; the broader the domain, the more difficult it becomes to cover with precise rules every possible combination of actions. Furthermore, smooth transitions to future states can only be achieved if the user provides an expected response; any deviation from the conversation design or complexity in the formulation of a turn will likely result in failure (managing multiple threads was however made possible to some extent in this paradigm by using a hierarchical structure with sub-automata (Klüwer, 2015, further details below).

#### 5.1.2 Statistical approaches

As for statistical approaches, methods that incorporate pretrained language models have the greatest potential to expand a model’s contextual understanding since they are able to represent complex states within a latent space. In recent years, large language models (LLMs) have made significant progress in natural language processing. A survey of capacities for such systems and how they work is provided by Wang et al. (2024). These systems can either be used in an end-to-end fashion or they can be applied to the modules of the standard dialogue system pipeline (i.e., natural language understanding, dialogue management, natural language generation). An end-to-end system can be prompted with conversation history, and can then generate possible developments in the conversation, i.e. the user responses. They are trained on a general purpose large corpus, and can be later fine-tuned for specific applications. A pre-prompt can be included in the input in order to specify the expected responses (style, length, allowed domains, etc.).

These systems aim to implicitly handle, in the way they work, two of the three problems identified: multi-intent detection and multi-thread management. Indeed, for these approaches, there are no assumptions about the content of the conversation in such a way that the system will generate a probable answer in relation to what it has already seen during its training. These approaches can take as input the history of the interaction, which is really suited to keeping track of the dialogue state (e.g. Wen et al., 2017; Ham et al., 2020; Imrattanatrai and Fukuda, 2023).

Although, generative AI is very impressive, it nevertheless suffers from inherent limitations. LLM’s ability to generalize, while a strength when it comes to understanding previously unseen contexts, can also be a double-edged sword, as generalizations can lead to hallucinations (e.g. Yamazaki et al., 2023). If the model encounters a question for which it has no answer, it may simply invent a response which shares characteristics of content it has seen in its training data, but which has no factual validity. There may also be issues with response consistency (i.e., asking the model the same question twice could result in different and/or contradictory responses, see Song et al., 2020; Kassner et al., 2021). For a task oriented system, it is very important to have precise control over the information delivered to the user and so steps must be taken to rein in its generation. Compensation mechanisms proposed in the literature include knowledge grounding (Lin et al., 2022; Sun et al., 2023) and fine-tuning (Nguyen et al., 2023). LLM’s are nevertheless difficult to restrain when the corpus used for training does not correspond exactly to the situation to manage. For example, an LLM could take the initiative and offer to accompany the user into the library, even though the robot is not equipped to move around independently. In a way, when describing what to do for every use case, thanks for instance to fine tuning or pre-prompting strategies, we come up against the same drawbacks as the ones of descriptive strategies.

A further drawback of LLMs is that the majority of them are trained on textual data alone, and when applied to managing embodied interaction, they will not take into consideration important multimodal features (e.g., intonation, gesture, laughter, etc.) which modify expectations about appropriate future turns (although there is increasing interest in incorporating such features, see e.g. Driess et al., 2023; Huang et al., 2023; Kharitonov et al., 2022, so these issues may be overcome in the near future).

### 5.2 Turn taking dynamics

A number of the observed failures could be better handled with improved turn taking skills. Pepper’s system relies purely on silence to detect the end of the user’s turn, which is clearly insufficient because it does not make the distinction between a within-turn pause (Harvey Sacks and Jefferson, 1974) and turn yielding. More sophisticated approaches incorporate a wider array of features to detect whether the user’s turn has come to completion. These signals include verbal, prosodic, breathing, gaze and gesture cues.

Verbal cues, including syntactic, semantic and pragmatic features, are important cues for human turn-end prediction (Ford and Thompson, 1996; Ford et al., 1996). Simple models that use only the part-of-speech of the final two words (Gravano and Hirschberg, 2011; Meena et al., 2014) are often able to detect incomplete turns as certain categories (e.g., determiners without the following noun) are unlikely to be the end of a contribution.^9^ In fact, leaving an utterance syntactically incomplete is a strategy employed by speakers in order to hold the floor (Selting, 2000).

As we saw in Section 4.3, syntactic completeness/incompleteness alone would be insufficient to capture all the failures present in our corpus. Large language models could provide richer syntactic, semantic and pragmatic information for end-of-turn prediction. Ekstedt and Skantze (2020) adapt a GPT-2 model to include transition relevance place (TRP) tokens as part of the model’s vocabulary and this method provides significant gains over simpler POS-based ones. Further progress could be achieved by incorporating knowledge of sequential context (see Section 4.3).

It has been an issue of debate whether prosodic cues are a necessary component of turn-end identification. Ruiter et al. (2006) removed pitch contours from recorded English conversations and this appeared to have little effect on human prediction accuracy. Bögels and Torreira (2015), however, found that prosody was crucial for distinguishing between holding an yielding at ambiguous TRPs. Ekstedt and Skantze (2022) found similar results.

Non-linguistic features can also improve prediction accuracy. Exhaling has been associated with turn yielding and inhaling with turn retention (Rochet-Capellan and Fuchs, 2014; Ishii et al., 2014). Gaze helps regulate floor management (Degutyte and Astell, 2021) as, for example, looking away and, then, doing a return gaze, are conducts involved in building multi-unit turns, holding the floor, and accomplishing floor transfer (Kendon, 1967; Goodwin, 1981). This can however be complicated by other factors such as focus on reference items or the presence of multiple participants (Jokinen et al., 2013; Bilac et al., 2017). Gesture also plays a role, as turn completion will often coincide with movement completion (Duncan, 1972).

An ideal system would be able to anticipate the end of their interlocutor’s turn, as humans do. Evidence for this comes from the fact that human’s typically begin their turn within 200 ms of the end of the previous turn (Levinson and Torreira, 2015). This is insufficient time to plan and execute a new turn, suggesting that the listener both projected the end of the speaker’s turn and planned their turn in advance. They are able to do this projection because the previous turn has created expectations about what the current turn should entail. To replicate this behaviour for human-robot interaction, Ekstedt and Skantze (2021) use Turn-GPT (Ekstedt and Skantze, 2020) to project the end of the user’s turn by generating several possible continuations for the speaker’s utterance. If a sufficient number of these continuations project an ending, the model would prepare to take the floor. This method offered an improvement over a silence baseline in terms of reduction of both speech overlap and extended silences.

In our corpus analysis, we also observe failures in grounding (i.e., where the fact that Pepper is processing an action of the user is not properly communicated to the user). When the user interprets Pepper’s silence as a failure of hearing or understanding, they are likely to start a new turn, which can result in overlapping speech and a misalignment of user and agent expectations about upcoming speech. Adding fillers to robot speech has been shown to have a positive effect on the perceived speediness of the agent (Wigdor et al., 2016).

### 5.3 Multi-intent detection

As we have seen in Sections 4.2, one turn by the user does not always correspond with one intention, which can be a major source of errors for a system designed/trained to only look for one intention per turn. Simple solutions, such as taking the top-k classes predicted by a single intent classifier do not yield high results (Xu and Sarikaya, 2013) and so specialized multi-label models have been developed (Qin et al., 2020; Natarajan et al., 2020; Cheng et al., 2023).

Kim et al. (2017) looked for overt lexical markers (e.g. conjunctions) to identify possible divisions within the user’s utterance; once the sentence was split, single intent classifiers could be applied to the sentence parts. This method is quite limited though, as multiple intents sentences are not always so neatly demarcated and sometimes treating them separately can have negative consequences (e.g., *Find Avatar/and/play it.*).

Other research has investigated the joint prediction task of multiple intent with slot filling (Gangadharaiah and Narayanaswamy, 2019; Qin et al., 2020; Natarajan et al., 2020; Song et al., 2022). This design acknowledges the possibility that a once mentioned entity in the utterance could relate to different intents. Song et al. (2022) make use of global corpus statistics to learn explicit dependencies between intents and slots. Because the task of the classification is more difficult in a multi-label setting, error propagation can be a concern. To mitigate this, Cheng et al. (2023) propose a scope sensitive model which filters out words that are not semantically related to the intent classes.

Attempts have been made to leverage semantic similarities between intent classes to improve classification accuracy (Xu and Sarikaya, 2013; Wu et al., 2021). So rather than finding a mapping between user utterances and indexed intent categories, these methods find overlaps in meaning between categories such as getWeather and getTime (e.g., both are asking to retrieve information). Xu and Sarikaya (2013) model class features by predicting combined intent labels. Wu et al. (2021) learnt an intent semantic space by extracting the semantic information present in the intent labels. They then project the utterance embedding into the intent space and use linear approximation to learn the linear combination of the intent basis. This method can be extended to unseen intents during training, although the fine-grained distinctions between unseen classes is imperfect.

Dealing with multiple intents, once identified, can raise other issues for the system if two separate first pair parts have been put forward: a decision must be made about which action to deal with first. Landesberger and Ehrlich (2019) propose a six part strategy to prioritize responses to multi-intent turns: 1) explicit sequence ordering (e.g., *First tell me when the party is, then phone my mother.*), 2) thematic dependency (i.e., where the accomplishment of one task is dependant on the prior accomplishment of the other), 3) urgency, 4) efficiency and 5) personal preference (Landesberger and Ehrlich, 2020, show that prosodic features are able to detect the level of urgency within multi-intent turns).

Landesberger and Ehrlich (2018) also investigated user strategies for managing situations where there has been a misunderstanding in one of the intents from a multi-intent turn. Users displayed different behaviours, either addressing the system’s question and then providing a correction or only addressing the correction. A well designed model must be able to handle both of these possible reactions.

### 5.4 Multi-thread management

In the most basic scenario for task-oriented dialogue, either the user or the system will initiate a *First Pair Part* and this will immediately be followed by a *Second Pair Part* (e.g., question/response). However, in practice other acts may intervene before the *Second Pair Part* is completed, as we saw in Sections 4.4 and 4.1. This presents challenges for dialogue management: the system must be able to 1) represent active threads and 2) make decisions about which threads to pursue and in which order, as well as which to abandon.

Multi-threaded dialogue can refer to either embedded sequences (e.g., clarification questions) or interleaved ones (i.e., utterances pertaining to different tasks that are intermingled). Proposals to handle multiple active strands have involved extendable graph representations of the ongoing discourse and/or task stacks, where the most recently active thread is prioritized but incoming utterances can still be linked to lower level threads present on the stack, or they can create their own branch (Rosé et al., 1995; Lemon et al., 2002; Klüwer, 2015; Papaioannou et al., 2018; Maraev et al., 2020). For example, Lemon et al. (2002) use a dialogue move tree to represent the dialogue state and an active node list to represent the order of the most recently activated threads. If the incoming input satisfies an update function for one of the nodes on the stack, it is attached to that node. Similarly, Klüwer (2015) propose a dialogue manager that represents conversation threads (implemented as supernodes) as having one of three conditions: active, paused and inactive. If a suitable transition from the currently active thread is found lacking, then either a paused or a new thread is activated and this selection is done by considering the topic, dialogue act and domain.

Shi et al. (2019) observe that certain user queries are often followed by related queries, the results of which can cause the user to return to the initial task (for example, asking to schedule a meeting on a given date, followed by a weather check for that same date, and then revising/updating the date for the meeting to a new date with more suitable weather). In order to anticipate the users’ needs and reduce redundancies in query formulation and bolster intent classification, they propose a model that predicts whether the user is likely to switch topics and if so, they proactively provide the information.

Other works have incorporated the sequences identified in conversational analysis into their system design. In the Natural Conversational Framework (NCF) (Moore and Arar, 2019), interactions are designed as expandable sequences which can accommodate expansions such as clarifications and repairs. Kunneman and Hindriks (2022) take the patterns outlined in NCF and develop a dialogue engine which keeps track of the status of sequences (complete/incomplete). Duran (2023) trained models to automatically annotate adjacency pair labels which can then be used by a dialogue manager to determine the next move.

Models for dialogue disentanglement have been developed to separate chat room, social media and forum threads where multiple participants are conversing in interwoven discussions (Liu H. et al., 2021; Li et al., 2023; Gu et al., 2021). This separation aids information extraction and summarization. State-of-the-art deep learning techniques have applied global discourse structure to accomplish the task, which would not be available when processing the discourse in a linear fashion, however earlier techniques (Kummerfeld et al., 2019; Zhu et al., 2021) that model reply-to relations between utterances using features such as time between utterances, word overlap and anaphoric links could be transferable to actional/sequential thread classification in dialogue systems.

Not all actions that have been started must necessarily be handled by the system as the changing interactional context may make them irrelevant. We saw this in Section 4.1 when a greeting (*Hello Pepper*) is immediately followed by closing sequence (*Have a good day*), making the expectation for a return greeting less pertinent. Janarthanam and Lemon (2014) implement a policy for discarding threads that are no longer relevant (Queue revision), although their model only considers the user’s geographical location in a city as criteria for thread elimination and not interactional phenomena.

End-to-end systems for task-oriented dialogue (Qun et al., 2020; Wen et al., 2017)) allow the model to learn the types of sequences that appear naturally in the training corpus (which could include both interleaved and embedded sequences) without the model designer having to specify them. While there is a good chance large language models would be able to handle multiple threads, to the best of our knowledge, this has not been tested empirically and this would likely be dependent on the size/memory of the model, as well as the number of mixed sequences available in the training data.

Finally, it is important that the dialogue manager signals to the user which thread is being attended to. If the system is responding to anything but the most recently activated one, this could be confusing for the user. When humans switch between topics, they make use of discourse markers (Heeman et al., 2005; Yang et al., 2008). And when they return to a pre-existing thread, humans frequently restore the previous context with repetition (Yang and Heeman, 2009).

### 5.5 Multimodal cues

In order to facilitate an understanding of context, cues beyond the verbal need to be taken into consideration. Visual cues are a rich source of information for interaction, however from a technical point of view there are still challenges to overcome in dissecting intricate, evolving scenes in order to make them interpretable to the robot system.

A first step is to identify the presence of humans in the environment which can be accomplished using human detection neural models (Marvasti-Zadeh et al., 2021). More demanding is maintaining a consistent representation of the identified people, which is necessary to manage the context history (i.e., how long have I been talking to this person and what have we talked about). Not having mastered this skill, Pepper would often reintroduce himself mid-conversation during our experiment.

In a public space, where people are constantly moving in and out of the scene, people monitoring is not an easy task. Tracking algorithms have been developed (e.g. Zhang et al., 2022; Cheng et al., 2024), but these can break down when a person momentarily leaves or is occluded from a camera’s view (Liu S. et al., 2021). If people can be tracked at a fairly reliable level however, then cues regarding their movements and proximity to the robot can be used to evaluate the type of engagement the human wants to engage in (e.g., a quick hello/goodbye vs. a prolonged conversation).

Active speaker detection models (Min et al., 2022; Liao et al., 2023; Alcázar et al., 2020) which use both audio and visual signals as well as speaker relations, could aid in the detection of overlapping speech. Dedicated models for such events have also been proposed using audio alone for diarization tasks (e.g. Bullock et al., 2020). Once detected, the robot system could allow for more time for the user’s turn with the knowledge that a silence may not indicate the end of a turn, but a repair.

The visual is also important for embodied interaction as gesture (e.g., a head nod or a thumbs up) can be interpreted as a conversational turn in its own right: neural network architectures have made great strides in recent years in recognizing these actions (Ji et al., 2020).

## 6 Conclusion

In this work, we have identified failure types within a video-recorded corpus of human-robot interaction and, using the tools of conversational analysis, we have offered explanations for such communication difficulties. Experiments were carried out using a task-oriented conversational agent implemented on the robot Pepper to welcome and guide visitors in a library. When designing Pepper’s scenario, we did not strictly follow the robot’s guidelines, i.e., we did not use the display screen as recommended.^10^ Rather, in this scenario, the user is expected to mostly rely on adapting human adjacency pairs and the interaction overall organization in the case of service encounters. In this manner, we compiled a corpus of in-the-wild human-robot interactions and identified concrete and typical cases of human behaviour and adaptations when confronted with such a system used in public.

This setup allows us to identify and deeply analyse some failures during interactions, taking the system’s inability to contextualize turns as a central point. Our study is particularly interested in the way the context of failures can be described through the sequential organization of interaction, and methods from computer science to enhance the dialogue management in this regard.

The system used in our experiment cannot for instance use syntax and pragmatic reasoning to determine when the speaker has finished the current turn based on constraints imposed by the previous turn. Nor can it keep track of the opening and closing of adjacency pairs, when for example two actions are performed within the same turn or when a user switches threads after an issue with grounding.

Our analysis is based on an interpretation of the conversation in all its dynamism and sequentiality, rather than on a more local one. When analysing failures in the analytic section, we accordingly showed that the cues that the robot needs to identify can only be understood with the knowledge that interaction is managed through a sequential organisation of expectancy. This underlines the central role of contextualisation in dialogue modelling.

Because these cues are purposely made visible by participants (drawing from human-human interaction), we propose that they can be objectified and computed. This raises the question of how to compute these in accordance with the sequential organization that we highlighted. We present this (based on CA theory) as a normative organization and not a rule set, which consequently cannot be easily emulated with a rule-based approach. As highlighted in the theoretical section (Section 2), some of the human-human interaction norms hypothetically elicited by the robot may be rejected or welcomed by the users as reified rules for participating with it.

Through our analysis, where we bring to light the sequential and actional bonds between (parts of) turns, we identify events as instances of classes we define incrementally, taking place over the course of time. Whether or not it is possible to have better results through a statistical approach with regards to sequencing remains to be tested.

Failures in our experiment stem from the system’s inability to contextualize turns. The ability of models to build a relevant and appropriate representation or contextualization from a signal (such as speech/text) has been a long-standing concern in AI. This issue has been addressed through descriptive or expert approaches, and more recently, through machine learning, where large language models have proven effective. After describing the advantages and drawbacks of each of these paradigms, we review the literature for each of the highlighted causes of failure, namely turn-taking, multi-intent identification, multi-thread handling and multi-modal understanding.

The most promising of the covered methods are those that offer flexibility and robustness when faced with a broad range of different contextual states and complex user inputs. The use of large language models in conversational agents has great potential to overcome the observed failures, if controls can be put in place to control their generation. In contrast, descriptive approaches are more deterministic and predictable but may struggle to adapt beyond the specific frameworks for which they were designed.

This study raises a number of questions and challenges for dialogue modelling. Some of the studied patterns have not yet been investigated in state-of-the-art models (to the best of our knowledge). This is the case for multi-thread management, for which an LLM end-to-end approach appears to have great potential. While these models are promising, for some specific problems, LLMs may struggle to capture the contextual meaning of underlying structures present in the corpus. This is likely to be particularly pronounced when the training corpora are not dedicated to human-robot interaction, which can introduce biases. The corpus we collected has been labeled for each of the failure types, such that it could be used to probe an LLM’s latent representations (for example, by testing its ability to maintain context across multiple conversational threads or correctly identify multiple intents in a single interaction). These probes could then contribute to the building of a more advanced and relevant conversational agent.

The described work has been conceived independently from the application domain. As HRI research identifies failure types of different kinds, there is a need to build test scenarios that could be used to evaluate specific devices or use cases. We advocate that an embodied HRI scenario should elicit the situations we presented, as the interactional outcomes that we analyzed are ubiquitous and therefore should be handled adequately.

## Data Availability

The raw data supporting the conclusions of this article will be made available by the authors, without undue reservation.

## Footnotes

1
https://qisdk.softbankrobotics.com/sdk/doc/pepper-sdk/index.html

2 ELAN (Version 6.7) [Computer software] (2023). Nijmegen: Max Planck Institute for Psycholinguistics, The Language Archive. Retrieved from https://archive.mpi.nl/tla/elan

3
http://icar.cnrs.fr/corinte/conventions-de-transcription/

4 Another issue here is the fact that the self-presentation performed by Pepper conflicts with the recognition displayed by the human. However, the recognition and accomplishment of “greetings alone” exchanges between acquainted people supersedes this issue (that is, treating the address term “Pepper” as a cue for such an activity distinction would be of very low importance).

5 In fact, by the categorization of Pepper as “a regular” vs. a service provider, and as evidenced by the additional cases (and particularly 220328_06A) where laughter follows the greeting produced in the move, it can be considered as a “successful failure” from the user perspective, as proposed in Section 2.3.

6 If the system matches the fact that a place is “looked for” but the system does not recognize the place, it can then initiate a repair. Then, by responding to the repair initiated by the robot, users may isolate the name of the place they are looking for, thereby easing the recognition on the second attempt. The “repair list”, thus, has a lower priority on place recognition.

7 Another interesting event is observed in excerpt 220324_79, another event is not mentioned here it is the laughter that accomplishes a type of sequence-closing third assessment often seen in HRI (see also excerpt 220321_06 in Section 4.4 or 220928_19 in this section for the exact same events). Handling such interaction between humans is another issue that is relevant with regard to sequentiality but we will not address this here. It is also a good example of specific HRI practices (Licoppe and Rollet, 2020).

8 Note that ideally, here, in this specific context, the wordlist containing “no thanks” should not be able to match at all, but this does not entail the rest of the comment as such this situation can be reproduced in other contexts, such as following an offer.

9 An exception to this principle does occur when the speaker is eliciting the listener to finish their utterance when they cannot think of the word (Clark and Wilkes-Gibbs, 1986).

10
http://doc.aldebaran.com/download/Pepper_B2BD_guidelines_Sept_V1.5.pdf
